# Detecting microsatellite instability in colorectal cancer using Transformer-based colonoscopy image classification and retrieval

**DOI:** 10.1371/journal.pone.0292277

**Published:** 2024-01-25

**Authors:** Chung-Ming Lo, Jeng-Kai Jiang, Chun-Chi Lin

**Affiliations:** 1 Graduate Institute of Library, Information and Archival Studies, National Chengchi University, Taipei, Taiwan; 2 Department of Surgery, Division of Colon and Rectal Surgery, Taipei Veterans General Hospital, Taipei, Taiwan; 3 Department of Surgery, School of Medicine, National Yang Ming Chiao Tung University, Taipei, Taiwan; Chunghwa Telecom Co. Ltd., TAIWAN

## Abstract

Colorectal cancer (CRC) is a major global health concern, with microsatellite instability-high (MSI-H) being a defining characteristic of hereditary nonpolyposis colorectal cancer syndrome and affecting 15% of sporadic CRCs. Tumors with MSI-H have unique features and better prognosis compared to MSI-L and microsatellite stable (MSS) tumors. This study proposed establishing a MSI prediction model using more available and low-cost colonoscopy images instead of histopathology. The experiment utilized a database of 427 MSI-H and 1590 MSS colonoscopy images and vision Transformer (ViT) with different feature training approaches to establish the MSI prediction model. The accuracy of combining pre-trained ViT features was 84% with an area under the receiver operating characteristic curve of 0.86, which was better than that of DenseNet201 (80%, 0.80) in the experiment with support vector machine. The content-based image retrieval (CBIR) approach showed that ViT features can obtain a mean average precision of 0.81 compared to 0.79 of DenseNet201. ViT reduced the issues that occur in convolutional neural networks, including limited receptive field and gradient disappearance, and may be better at interpreting diagnostic information around tumors and surrounding tissues. By using CBIR, the presentation of similar images with the same MSI status would provide more convincing deep learning suggestions for clinical use.

## Introduction

Colorectal cancer (CRC) affected more than 1.9 million people in 2020 and was responsible for approximately 935,000 deaths. It is currently the second leading cause of death and the third most commonly diagnosed cancer worldwide [1]. Microsatellite instability-high (MSI-H) is a defining characteristic of hereditary nonpolyposis colorectal cancer syndrome, and around 15% of sporadic colorectal carcinomas exhibit MSI-H [2]. Tumors with MSI-H display distinct features, such as a preference for developing in the proximal colon, lymphocytic infiltration, and a poorly differentiated, mucinous, or signet ring appearance [2,3]. Furthermore, MSI-H tumors are associated with specific pathological characteristics, such as a host immune response, including Crohn’s-like lymphoid reaction, intratumoral lymphocytic infiltrate, and intraepithelial T cells. Compared to MSI-low (MSI-L) and microsatellite stable (MSS) tumors, CRCs with MSI-H have a more favorable prognosis [2,4].

The identification of MSI in CRC has revealed the heterogeneity of this disease, and the use of neoadjuvant therapy with immune checkpoint blockade in dMMR/MSI-H tumors has resulted in favorable response rates. This has significant clinical importance for organ-preserving approaches [5–7] and has implications for the treatment strategy in the management of CRCs. As a result, MSI testing has become a crucial component of CRC management. The Bethesda guidelines [8] have been widely accepted as the criteria for MSI testing. Currently, the NCCN Guidelines recommend universal MMR or MSI testing for all patients with a personal history of colon or rectal cancer. Besides serving as a predictive marker for immunotherapy in advanced CRC, MMR/MSI status can also aid in identifying individuals with Lynch syndrome [9].

Various methods for detecting microsatellite instability include fluorescent multiplex polymerase chain reaction (PCR) and capillary electrophoresis (CE) [10,11], immunohistochemistry (IHC) [12], and next-generation sequencing [13]. However, these techniques require a considerable amount of resources and labor. Some studies have examined the usefulness of histopathology in identifying MSI-H cancers by evaluating the pathologic features. While histopathological evaluation can be used to prioritize sporadic colon cancers for MSI studies, the morphological prediction of MSI-H has low sensitivity, necessitating molecular analysis for therapeutic decisions [14].

Colonoscopy is a valuable tool for diagnosing CRC, providing important information about the appearance, location, and depth of invasion of tumors in the colon wall. Tumors can vary in their appearance, with irregular, depressed, or ulcerating surfaces, and surrounding tissues can also provide important information. Statistical analysis can be used to summarize image characteristics that differentiate between different tumor statuses, but quantifying these image findings can be challenging. The features used to describe CRC status can be complex and difficult to quantify, and changes in illumination can result in changes in the findings. Additionally, interpreting the relationship between tumors and adjacent tissues can be difficult. Machine learning classifiers have been developed that use various methods to combine image features to classify tumors, providing an overall evaluation by probability and solving the problem of considering numerous findings simultaneously.

Deep learning offers an improved approach for extracting image features, as it can map image pixels into a high-dimensional feature space and automatically interpret the relevant image characteristics for a specific classification task without the need for human intervention. In the field of computer vision, deep learning architectures such as convolutional neural networks (CNNs) and vision Transformers (ViTs) have been developed. While CNNs have been used for pattern recognition in colonoscopy, they have limitations in scaling up the receptive field. This study proposed the use of ViT, which considers the global relationships between tumors and adjacent tissues in colonoscopy, as a more promising approach for feature extraction.

Echle et al. [15] used haematoxylin and eosin (H&E)-stained slides and molecular analysis findings to validate CNN approaches for predicting MSI in colorectal tumors across all stages. They achieved a mean area under the receiver operating characteristic curve (AUC) of 0.92. Yamashita et al. [16] proposed another CNN approach, based on a modified MobileNetV2 architecture pre-trained on ImageNet, and fine-tuned it to detect MSI from H&E-stained whole-slide images. Chang et al. [17] further improved on this by adding an attention mechanism to the CNN, achieving an AUC of over 0.95 in predicting MSI in H&E-stained images. Peng et al. [18] classified different tissues in colorectal cancer histology slides using a CNN-based image retrieval approach, which provided more transparency and generalizability, and achieved higher precision than a classification network. Finally, Komura et al. [19] proposed a CNN-based content-based image retrieval (CBIR) method that successfully predicted 309 combinations of genomic features and cancer types by retrieving histologically similar images.

Previous studies have shown the diagnostic potential of H&E-stained slides in detecting MSI, with researchers from various countries using CNN-based methods to establish prediction models and investigate image patterns. In contrast, our study utilized the ViT architecture instead of CNNs to predict MSI, benefiting from multi-head self-attention to address the limitations of CNNs in scaling up the receptive field and avoiding gradient vanishing [20]. Furthermore, this study serves as a proof of concept, highlighting the distinctive aspect of our research, which utilizes colonoscopy instead of H&E-stained slides for deep learning-based MSI prediction. We also implemented CBIR to demonstrate its precision in identifying similarities and differences among tissue types, which can aid decision-making and establish correlations between classification bases and tissue types.

## Materials and methods

### Study population

In this research, we conducted an analysis of two cohorts consisting of CRC patients. The first cohort was enrolled between May 2014 and December 2017 and comprised of 441 patients, among whom 407 had MSS CRC and 34 had MSI-H CRC. To increase the sample size of MSI-H CRC, we enrolled an additional 89 patients who underwent surgery between January 2018 and May 2021. All patients underwent primary tumor resection at our hospital, and preoperative colonoscopy imaging was conducted for analysis. The primary tumor colonoscopy images were randomly captured during tumor diagnosis or preoperative localization. Patients with synchronous or metachronous CRC and those who received neoadjuvant therapy were excluded from the study. Patients who didn’t have data about MSI status or pre-operative colonoscopy images were also excluded. The institutional review board approved the study protocol (2023-01-001CC), and the requirement for written informed consent was waived. The data were collected and analysis was conducted since Feb 2023 after approval of institutional review board. Because the correlation of MSI and the colonoscopy is necessary in the current study, the authors (J-K Jiang and C-C Lin) could access to information that could identify individual participants during or after data collection.

### MSI testing

MSI testing was performed at our hospital since 2014. Immunohistochemistry (IHC) staining of tumor tissue was used to detect the expression of the four mismatch repair (MMR) genes, namely MLH1, MSH2, MSH6, and PMS2. A normal IHC test indicated that all four MMR proteins were expressed normally, and the tumor was considered MSS. Conversely, an abnormal IHC test suggested that at least one of the MMR proteins was not expressed, indicating a possible inherited mutation in the related gene. Loss of protein expression by IHC in any of the MMR genes was confirmed by specialized gastrointestinal pathologists with expertise in CRC pathology.

### Vision Transformer

**Fig 1:**
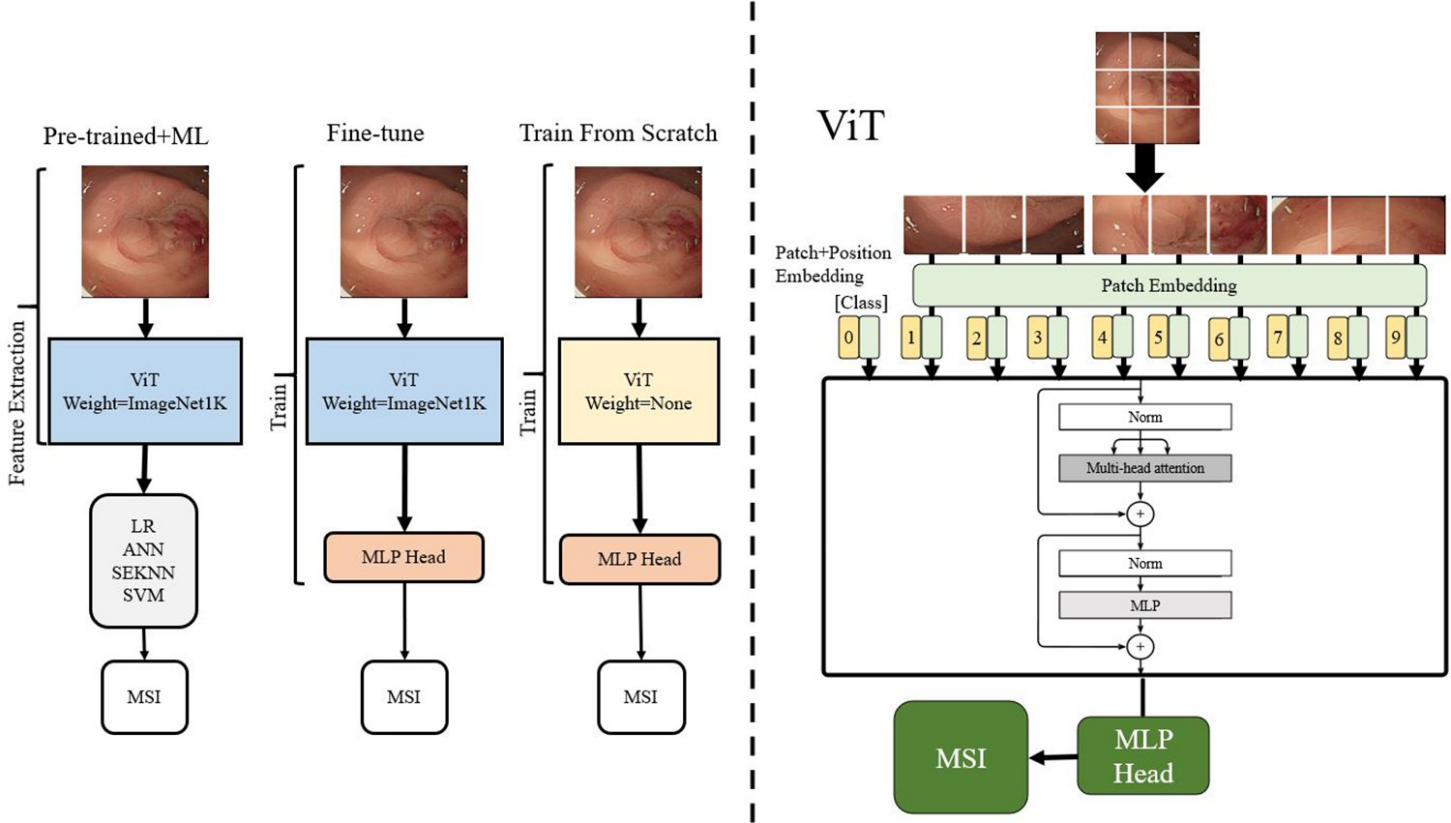
Vision Transformer modeling.

Deep learning approaches including CNNs and ViT have been suggested to recognize patterns in medical images [21–23]. Especially, ViT has shown improved generalization compared to CNNs, meaning that it can perform well on images outside of the training dataset [24,25]. ViT’s architecture, as depicted in Fig 1, begins by flattening the split patches and projecting them into patch embeddings.

This resulting sequence is then preprocessed with a prepend class token (*x*_*class*_) and position embeddings (*E*_*pos*_) to preserve the positional information of the original image, as expressed in Formula 1:

$$z_{0}=[x_{class};x_{p}^{1}E;x_{p}^{2}E;\ldots ;x_{p}^{N}E]+E_{pos}$$ (1)

where *x*_1_, *x*_2_, …, and *x*_*p*_ are patches.

Subsequently, the encoder is constructed using multiple rounds of concatenating multiheaded self-attention, and multilayer perceptron blocks. The blocks are layered in the usual way and concluded with a residual connection. The multihead self-attention mechanism employed by ViT involves converting the input *x*^*i*^ into *a*^*i*^, which is then processed through the self-attention layer. At this stage, it is multiplied by three different matrices to generate query (*q*), key (*k*), and value (*v*) vectors. These vectors can be further expanded into a multihead structure by repeating the operation. The *q* vector is then used to perform inner products with different *k* vectors, producing similarity measurements that consider long-term dependence. The resulting weights are then multiplied by the *v* vector to produce the final output.

### Model training

Training deep neural networks involves optimizing the model parameters to minimize the loss function for a specific task. There are three different approaches as shown in Fig 1 to model training, each with trade-offs between accuracy and efficiency: training from scratch, pre-training, and fine-tuning. Training from scratch initializes the training process with random weights and biases, and the model is trained on a large labeled dataset for a specific task. While computationally expensive, this approach can achieve good performance with enough labeled data. Pre-training involves training a neural network on a large and diverse dataset, such as ImageNet, to generate substantial weights and biases that represent characteristics of the data. If the labeled dataset for the specific task is limited, pre-training can provide general features that help distinguish data. Fine-tuning continues training on a smaller, task-specific dataset to learn more specific features related to the target task, leading to faster convergence and better performance. Fine-tuning requires fewer computational resources and is faster than training from scratch. In the experiment, using ViT as the base deep learning architecture, the three approaches were implemented for comparisons for the MSI prediction.

In the literature, when trained on large-scale datasets, ViT outperformed the ResNet architecture [20]. Thus, the pre-trained parameters used in the experiment was trained from ImageNet ILSVRC 2012 dataset [26], containing 1.3M images and 1k classes. By splitting the input image with patch size = 16×16, the multi-head self-attention mechanism trained 768 feature vectors to represent image characteristics.

### Performance evaluation

The approaches of train from scratch and fin-tune are complete neural networks which have the end layer to generate classification labels for each image. For a specific task, the output vector represents the probabilities of the input belonging to each class. Softmax function can normalize each element in this vector to a range of 0 to 1, and the sum of probabilities is 1. Consequently, the softmax function can interpret the probability vector output as the probabilities for each corresponding class. During training, the cross-entropy loss function calculates the difference between the predicted probabilities and the true labels, and then the backpropagation mechanism is used to update the weights in the network.

Pre-training approach used an alternative way for classification since it is trained based on the prior data and labels which can’t used in other tasks. Extracting features from the pre-train model and combining them in machine learning classifiers would be more practical. Additionally, the machine learning classifiers can perform better in generating nonlinear decision boundaries than softmax. Also, the minority class can be effectively handled in the classification.

In the experiment, four classifiers were used for training and testing the MSI classification using pre-train features, including logistic regression (LR) [27], artificial neural network (ANN) [28], subspace ensemble k nearest neighbor (SEKNN) [29], and support vector machine (SVM) [30]. The performances of different classifiers were calculated and compared in the experiment.

LR uses a logistic function, also known as the sigmoid function, as the cost function. The sigmoid function is an S-shaped curve that maps any number to a score between 0.0 and 1.0. In LR, the goal is to predict the probability of a binary outcome based on one or more input features. The model assigns weights and intercepts to each feature, and then uses these weights and intercepts to compute a score for each data point. The score is transformed into a probability value using the logistic function, which maps the score to a value between 0.0 and 1.0.

In ANN, the network is composed of layers of interconnected nodes, or neurons. The input features are fed into the network, and each feature is individually connected to the neurons in the middle hidden layer with different connection strengths, which are represented by weights. The neurons in the hidden layer then process the inputs and pass their outputs to the next layer until the output layer is reached, which produces the final output of the network. The backpropagation calculates the error between the predicted output and the true labels and uses this error to adjust the weights of the connections between the neurons. By iterations, the network is able to learn and improve its predictions.

SEKNN is an ensemble method that utilizes random selection to generate subsets of the original features. By creating multiple models based on these subsets, the approach combines models with different feature sets that provide diverse perspectives on the data, resulting in better training. This method is particularly useful for k nearest neighbor (KNN) since KNN is sensitive to changes in features. The SEKNN method is based on the using of multiple KNN models, which can help overcome the limitations of a single model trained on the entire feature set.

SVM is used due to its effectiveness in high-dimensional spaces, meaning it can handle data points that have many features or dimensions. SVM works by finding the hyperplane that best separates two classes in the feature space. This hyperplane is selected to maximize the margin, which is the distance between the hyperplane and the nearest data points from each class. In SVM, a kernel function is used to transform the input features into a higher-dimensional space where a linear decision boundary can be identified. By mapping the original feature space to this higher-dimensional space, the algorithm can find a decision boundary that is capable of separating the data points into different classes.

The MSI value of each case was predicted based on the trained models. The resulting probability was used for binary classification using a threshold, where the patient was classified as either having MSI-H or not. To evaluate the generalization ability of the model, five-fold cross-validation was employed. The dataset was divided into five equally sized groups, with each group being used once as a test set while the remaining nine groups were used for training. This process was repeated five times, and the results were averaged to obtain the final performance. Performance indices, including accuracy, sensitivity, specificity, and the area under the receiver operating characteristic curve (AUC), were used to evaluate the models’ performances. AUC was used to consider the trade-offs between sensitivity and specificity at different thresholds [31].

### Image retrieval

A CBIR system can automatically extracts the characteristics of a query image and compare them to the existed target image database to obtain interested images in an objective and rapid way. The performance evaluation of a CBIR system is reliant on its ability to retrieve images in a rank order that corresponds to the similarity between the query image and the images in the database. The ranking is determined by measuring the similarity between the query image and the target images. To measure the effectiveness of the CBIR system, the ground truth relevance images of the targets must be labeled. The ratio of relevant images to retrieved images is used to establish the benchmark for the top k accuracy, which indicates the number of relevant images retrieved in the top k. In the experiment, multiple query images were used to test the CBIR system, and the mean average precision (mAP) was calculated for each top k cutoff [32]. TP means the total relevance of the top k. FP means the total irrelevance of the top k.


$$TP=\sum_{n=1}^{k}R_{n}$$ (2)

$$FP=\sum_{n=1}^{k}{(1-R}_{n})$$ (3)

$$Precision=\frac{TP}{\left(TP+FP\right)}$$ (4)

$$AP=\frac{1}{\left|R\right|}\sum_{k=1}^{\left|R\right|}Precision(R_{k}),mAP=\frac{1}{Q}\sum_{k=1}^{Q}{AP}_{k},Q:query$$ (5)

## Results

**Fig 2:**
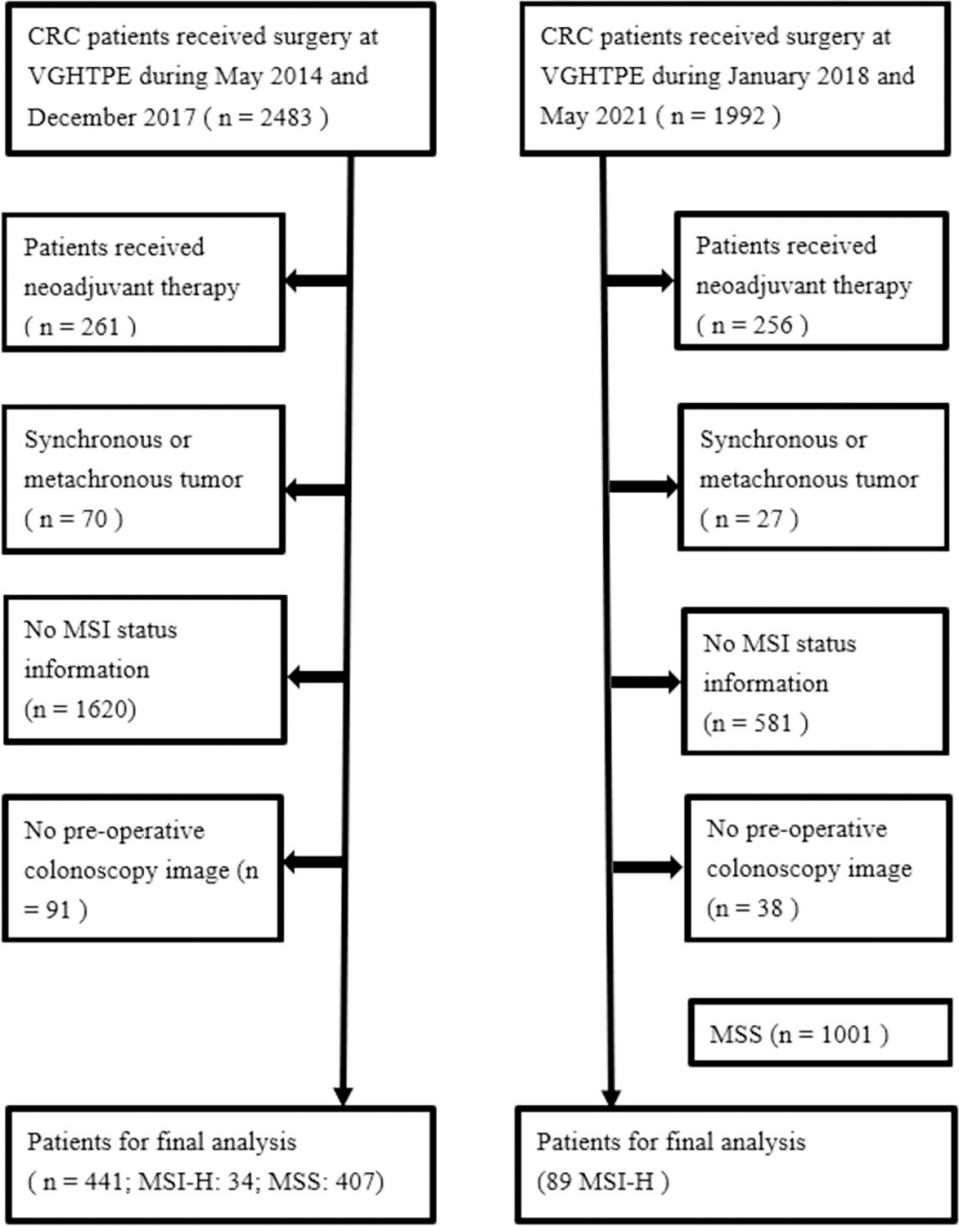
Flow diagram for the patients enrolled in the current study.

**Fig 3:**
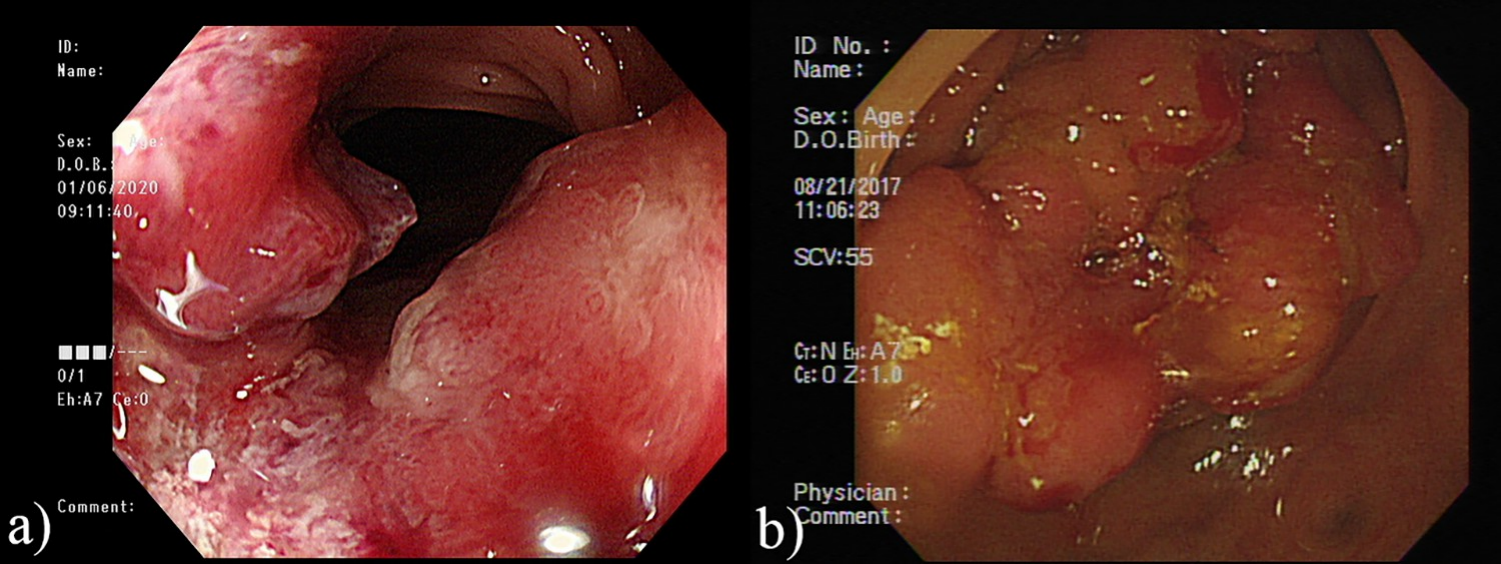
Demonstration of colonoscopy images of colon cancer with different MSI status. (a) MSI-H (b) MSS.

**Fig 4:**
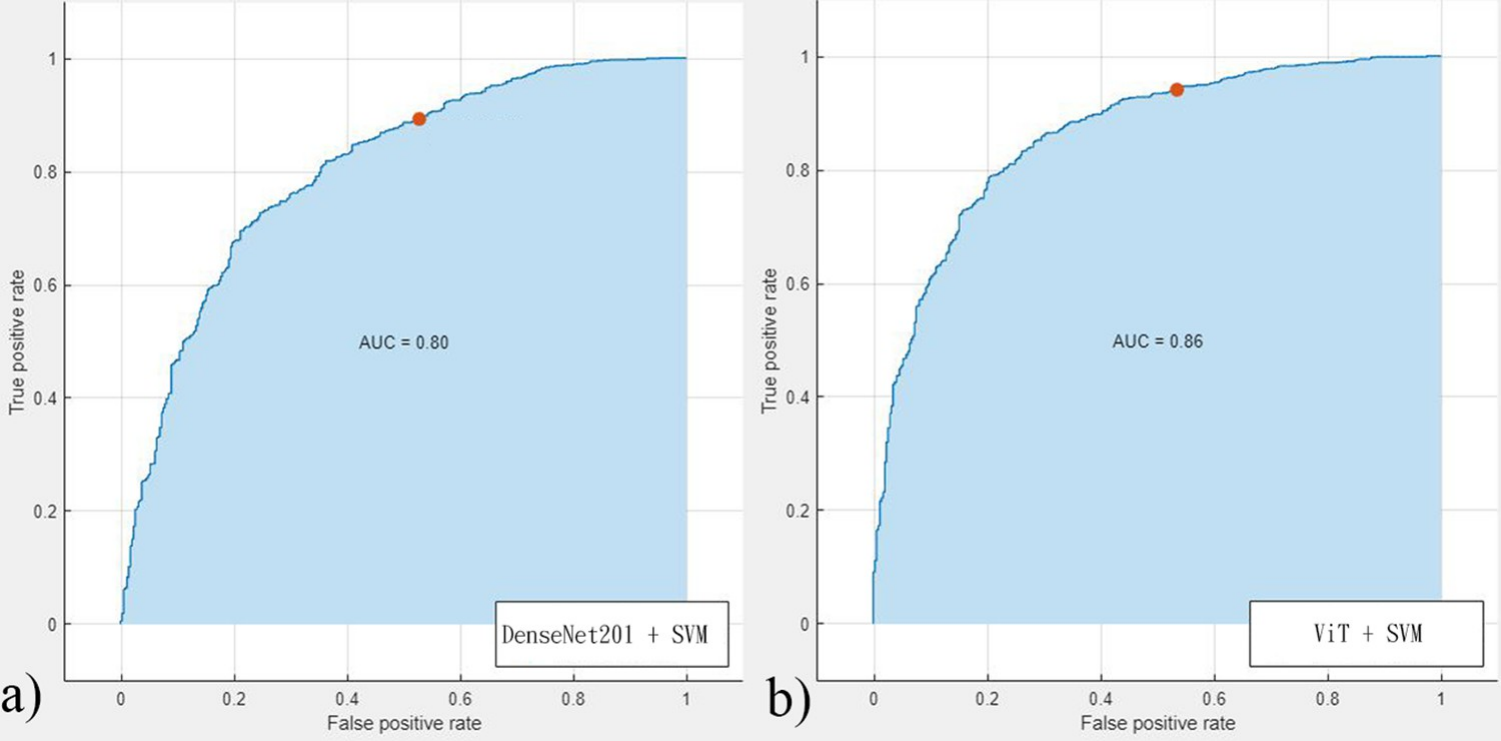
Receiver operating characteristic curve of different features combined in SVM to MSI prediction. (a) DenseNet201 features (b) ViT features.

This study enrolled a total of 123 MSI-H tumors and 407 MSS tumors, which comprised 427 MSI-H and 1590 MSS colonoscopy images. Fig 2 demonstrate the flow diagram for the patients enrolled in the current study. Table 1 provides a summary of the clinicopathologic characteristics of MSI-H and MSS tumors. When compared to MSS tumors, MSI-H tumors exhibit significant differences, including earlier staging, a predominant occurrence on the right side, higher rates of poor to undifferentiated grading, increased presence of tumor with ≥50% mucin component, elevated occurrences of lymphovascular invasion (LVI), perineural invasion, and signet ring cell components. Additionally, there is a tendency for these MSI-H tumors to be more prevalent in females (p = 0.066). Fig 3 demonstrate the gross images of MSI tumor and MSS tumor. To conduct the image retrieval process, the collected images were separated into the target image database (80%) and query images (20%). The similarity measurements between the query images and the target image database were based on image characteristics trained from the target image database, and the training involved five-fold cross-validation. Table 2 displays the classification performances of various learning networks, including DenseNet201, which was compared to ViT. Overall, the approaches of ViT were superior to DenseNet201. Among the approaches, combining pre-trained features in SVM outperformed the ways of fine-tuning and training from scratch. Based on the performance comparisons in Tables 3 and 4, SVM was selected as the best machine learning classifier. Fig 4 illustrates the receiver operating characteristic (ROC) curve and AUC value of the best performance achieved by DenseNet201 and ViT.

**Table 1:** Clinicopathologic profile of all colorectal cancer patients.

Characteristic (number)	MSI-H (123)	(%)	MSS (407)	(%)	p
Age Mean ± SD	66.7±15.0		63.9±12.6		0.067
Age ≧50 y/o Yes No	105 18	85.4 14.6	355 52	87.2 12.8	0.594
Gender Male Female	61 62	49.6 50.4	240 167	59.0 41.0	0.066
PreOP CEA level (ng/mL) Mean ± SD	11.7±27.1		31.7±191.6		0.248
Elevated PreOP CEA≧5 ng/mL Yes No	41 82	33.3 66.7	169 237	41.5 58.2	0.221
Stage I II III IV	25 69 23 6	20.3 56.1 18.7 4.9	83 124 135 65	20.4 30.5 33.2 16.0	0.001
Location Right-sided colon Left-sided colon Rectum	84 28 11	68.3 22.8 8.9	104 218 85	25.6 53.6 20.9	0.001
Grade of differentiation Well to moderate Poor to undifferentiated	98 25	79.7 20.3	378 29	92.9 7.1	0.001
Mucinous component ≧50% < 50%	25 96	20.7 79.3	19 384	4.7 95.3	0.001
LVI Yes No	18 99	15.4 84.6	119 257	31.6 68.4	0.001
Perineural invasion Yes No	4 113	3.4 96.6	47 329	12.5 87.5	0.005
Signet ring cell component Yes No	13 104	11.1 88.9	8 368	2.1 97.9	0.001*

LVI: Lymphovascular invasion.

*Fisher’s Exact Test.

**Table 2:** Classification performances of different learning networks.

	Accuracy	Sensitivity	Specificity	PPV	NPV	AUC
DenseNet201 (train from scratch)	51%	85%	42%	30%	93%	0.75
DenseNet201 (fine-tune)	77%	64%	80%	48%	89%	0.80
DenseNet201 (pre-train+ML)	80%	47%	89%	55%	86%	0.80
ViT (train from scratch)	69%	57%	72%	42%	87%	0.74
ViT (fine-tune)	81%	49%	89%	56%	86%	0.77
ViT (pre-train+ML)	84%	47%	94%	68%	87%	0.86

**Table 3:** Performance indices of combining DenseNet201 features in machine learning classifiers.

Classifier	Accuracy	Sensitivity	Specificity	PPV	NPV	AUC
LR	55%	51%	56%	24%	81%	0.53
ANN	79%	50%	87%	51%	87%	0.74
SEKNN	78%	43%	87%	48%	85%	0.67
SVM	80%	47%	89%	55%	86%	0.80

**Table 4:** Performance indices of combining ViT features in machine learning classifiers.

Classifier	Accuracy	Sensitivity	Specificity	PPV	NPV	AUC
LR	72%	52%	78%	39%	86%	0.69
ANN	81%	52%	89%	56%	87%	0.80
SEKNN	82%	46%	92%	62%	86%	0.77
SVM	84%	47%	94%	68%	87%	0.86

**Fig 5:**
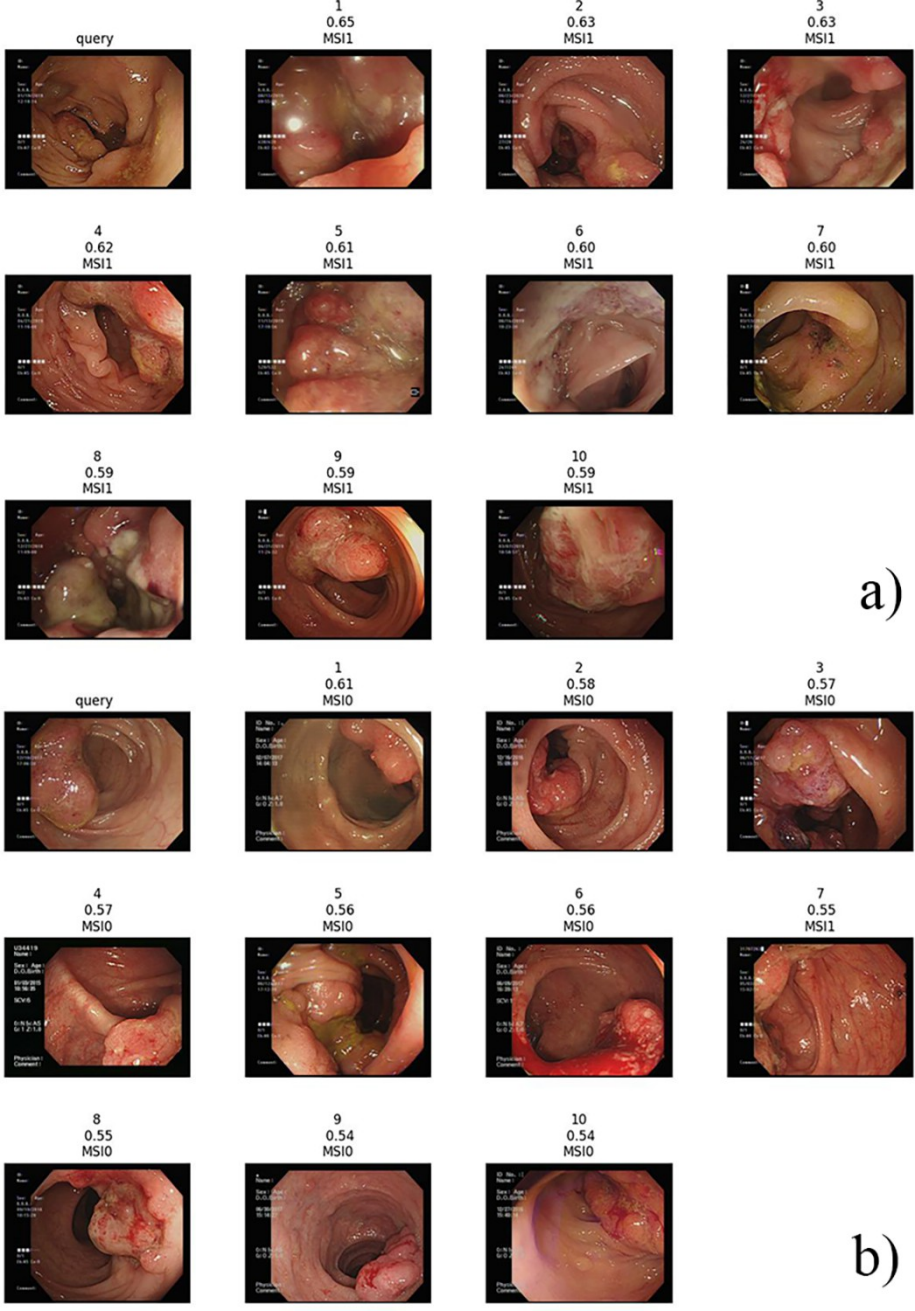
Retrieved top 10 colonoscopy images using ViT fine-tune features (a) the query of MSI-H (b) the query of MSS.

Table 5 lists the top-10 image retrieval result. Based on the trained features in the classifications, ViT features had mAP = 0.81 which outperformed DenseNet201 features having mAP = 0.79. ViT still have better result of image retrieval than DenseNet201. Fig 5 shows the top-10 image retrieval results of the queries of MSI-H and MSS images based on ViT fine-tune features.

**Table 5:** Retrieval performances of different learning networks.

	Top-10
DenseNet201 (pre-train)	0.59
DenseNet201 (fine-tune)	0.79
ViT (pre-train)	0.72
ViT (fine-tune)	0.81

## Discussion

Apart from certain clinical and pathologic characteristics, MSI status can also serve as a biomarker for anticipating the response to particular therapies. MSI status is particularly useful in guiding treatment decisions for stage II colorectal cancers, and MSI-high tumors have demonstrated a high degree of responsiveness to immune checkpoint inhibitors (ICI) [4]. As a result, MSI testing is a recommended component of the standard of care for all patients diagnosed with CRC [33]. Numerous studies have employed deep learning for the prediction of MSI status, with the majority of them using histopathologic images [34–44]. Additionally, radiomic signatures have been demonstrated to predict genetic alterations [45–50]. The goal of this study is to utilize deep learning models for the prediction of MSI status through the image retrieval of colonoscopy images of primary tumors. Colonoscopy is a widely used and convenient method for evaluating tumor status in clinical examinations. It has the advantage of presenting real-time image information, which can reduce time and costs compared to using histopathologic images and radiomic signatures. To our knowledge, this is the first study utilizing colonoscopy images for the prediction of MSI status in CRC.

By utilizing various training methods, the ViT architectures demonstrated superior performance compared to DenseNet201, which is another type of CNN architecture. One possible reason for this is that the attention mechanism used in ViT enables it to better analyze tumors and surrounding tissues through similarity measurements between image patches. SVM was utilized in this study to combine deep learning features, and the receiver operating characteristic curve for DenseNet201 and ViT features were 0.80 and 0.86, respectively. The NPVs were approximately 86–87%, suggesting a potential reduction in the expenses associated with MMR testing in routine clinical practice. Furthermore, leveraging pre-trained features enhances the efficiency and practicality of using deep learning.

The current study has several limitations. Firstly, the number of patients enrolled is limited, and only a small percentage (7.7%) of patients in the initial cohort had MSI-H tumors. To address this, we enrolled another MSI cohort, but this may impact the clinical applicability of the current model in real-world settings. The next step should be to perform further external validation using multicenter patients to generalize the clinical utility of the model. As more data becomes available, both training from scratch and fine-tuning methods are likely to yield better results. However, the increased amount of data also means that computational resources and training time will be more demanding. Secondly, we omitted the inclusion of family history, a crucial parameter in clinical Lynch syndrome assessment. Additionally, we did not incorporate clinicopathological features like age, staging, tumor location, and tumor differentiation into our predictive model, even though they may have predictive value for MSI status. A future study should aim to integrate additional diagnostic information to improve the accuracy of the model. The use of image retrieval to distinguish between various genetic backgrounds, such as sporadic or hereditary MSI-H, has not yet been fully explored. This aspect would be a subject of further investigation in our study. Third, further MLH1 methylation status and/or genetic testing, such as, next-generation sequencing is needed in patients with the loss of one or more MMR markers to differentiate sporadic MSI-H patients from Lynch syndrome. However, we didn’t have this information. How the background of MSI-H tumor affects the decision of ViT features remained elusive. To tackle this concern, it is necessary for us to initiate a prospective study to acquire this information and establish more robust evidence for practical application. In addition, we utilized IHC for MMR proteins to detect the MSI status, rather than PCR-based. IHC for MMR proteins is the initial step to screen for Lynch syndrome [51]. Previous studies had proven to reveal a high coincidence rate of the two methods for detecting MSI status up to more than 90% [52–54]. IHC and the PCR method had high consistency in MSI status. Compared with PCR, the IHC method is the preferred single screening test and is more economical and more convenient for clinical operations. While there have been studies aimed at distinguishing MSI-H CRCs from MSS CRCs based on differences in their histopathological and pathomorphological characteristics, such as the prevalence of mucinous adenocarcinoma and aggressive histological features in MSI-H tumors [55], there is currently no evidence suggesting that colonoscopy can grossly differentiate these features. However, it remains uncertain whether colonoscopy can successfully identify these distinct characteristics.

This study proposed the utilization of pre-trained ViT features to achieve a rapid and substantial MSI classification outcome, which is crucial for clinical applications. In addition to a numerical value indicating the MSI classification, the study also presented additional evidence through an image retrieval method, similar to the approach suggested by Komura et al. [19] in their study. Through a top-10 image display, physicians can observe whether images with similar compositions possess the same MSI classification, thereby increasing confidence in the decision-making. A future research direction could be to investigate the image differences among various MSI statuses. Similar to previous studies utilizing radiomic signatures [45–50], discovering more evidence from a larger pool of colonoscopy images would enhance the results’ credibility and enable widespread use in clinical settings.

## Conclusions

CRCs characterized by MSI-H have a more favorable prognosis. In this study, to achieve a rapid and cost-effective outcome with limited cases, we proposed the use of ViT features extracted from colonoscopy images. By combining pre-trained features in SVM, the classification results exhibited 84% accuracy and an AUC of 0.86. Compared to conventional CNNs, ViT based on the patch embedding and self-attention mechanism addresses the issue of limited receptive fields and gradient disappearance. The experiment also presented a CBIR result, with a mAP of 0.81, illustrating that images with similar content have the same MSI status. This proposed image classification and retrieval procedure, in conjunction with colonoscopy examination, makes MSI prediction more accessible and convenient for clinical use.

## Supporting information

S1 Checklist STROBE statement—Checklist of items that should be included in reports of observational studies. (DOCX)

S1 File Supporting information zip file contains image features. (ZIP)
